# An Unusual Electrogram Sequence with a Questionable Potential on the His-bundle Catheter During Sinus Rhythm: What Is the Mechanism?

**DOI:** 10.19102/icrm.2022.130305

**Published:** 2022-03-15

**Authors:** Ahmet Lutfu Sertdemir, Meryem Kara, Serdal Bastug, Bulent Deveci, Ahmet Korkmaz, Ozcan Ozeke, Serkan Cay, Firat Ozcan, Serkan Topaloglu, Dursun Aras

**Affiliations:** ^1^Department of Cardiology, University of Health Sciences, Ankara City Hospital, Ankara, Turkey; ^2^Department of Cardiology, Necmettin Erbakan University Meram Medical Faculty, Konya, Turkey; ^3^Department of Cardiology, Ankara Yıldırım Beyazıt University School of Medicine; Ankara City Hospital, Ankara, Turkey

**Keywords:** Late potential, LAVA, periaortic fibrosis, periaortic scar, signal analysis, ventricular tachycardia

## Abstract

The presence of 2 ventricular signals caused by structures near the His bundle region is rare. Some associative and dissociative maneuvers for dissociating a certain electrical signal from others of known origin are used to ascertain the source of an unknown potential.

A 64-year-old man with recurrent episodes of wide QRS tachycardia with the absence of overt structural heart disease underwent an electrophysiological evaluation. An echocardiogram showed mildly depressed left ventricular function. During an electrophysiological study, the electrogram in **Figure 1** was obtained. The arrow points to a signal after the ventricular potential on the His-bundle (HB) catheter that was consistently seen in sinus rhythm **(Figure 1)**. What could be the origin of this second unexpected potential?

## Discussion

The presence of 2 ventricular signals caused by structures near the HB region is rare.^1,2^ A wide range of diagnoses are possible, including (1) intermittent accessory pathway (AP) conduction with an antegrade 2-for-1 phenomenon, (2) intermittent fused premature ventricular complexes (PVCs) arising from the diseased tissue near the HB region, (3) intermittent right bundle ectopy, (4) intermittent nodoventricular (NV) conduction, (5) intermittent atriofascicular (Mahaim fiber) conduction, (6) fragmented ventricular potential, or (7) basal septal or periaortic ventricular late potentials and local abnormal ventricular activities (LAVAs).^1–3^ Mahaim fibers or the NV pathway may also produce a second HB deflection after the ventricular potential on the HB catheter because of the retrograde conduction of the right bundle.^2^ Other antegrade-conducting unusual AP connections from the right atrial appendage to the right ventricular outflow tract may also cause complex signals on the HB catheter. A 2-for-1 phenomenon can occur when the atrial impulse simultaneously travels down the AP (activating the annular ventricular myocardium) and the atrioventricular node (AVN) and the HB (activating the relatively more apical ventricular myocardium, close to the exit site of the right bundle). Afterward, the ventricular activation wavefront may travel back toward the base (2-for-1 signals can also occur with dual AVN physiology or with anteriorly and basally located APs).^1^

**Figure 2:**
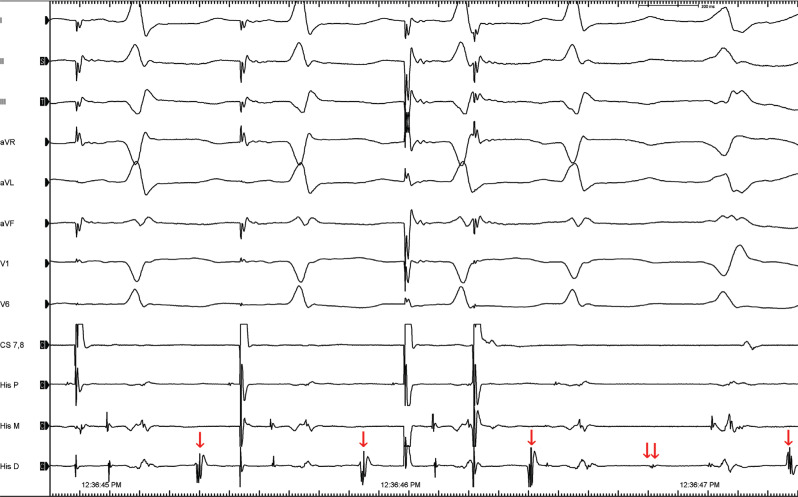
Programmed atrial stimulation shows that AH jumps with further delay (double arrow) in activating the tissue responsible for the unexplained potential (single arrows) after S2.

**Figure 3:**
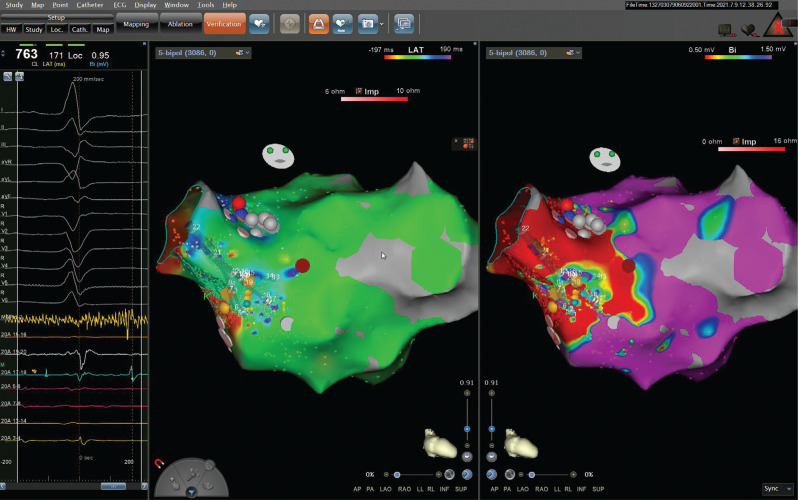
Isolated periaortic scar with a local abnormal ventricular activity on a local His potential electrogram during sinus rhythm recorded in a patient with the absence of overt structural heart disease.

**Figure 4:**
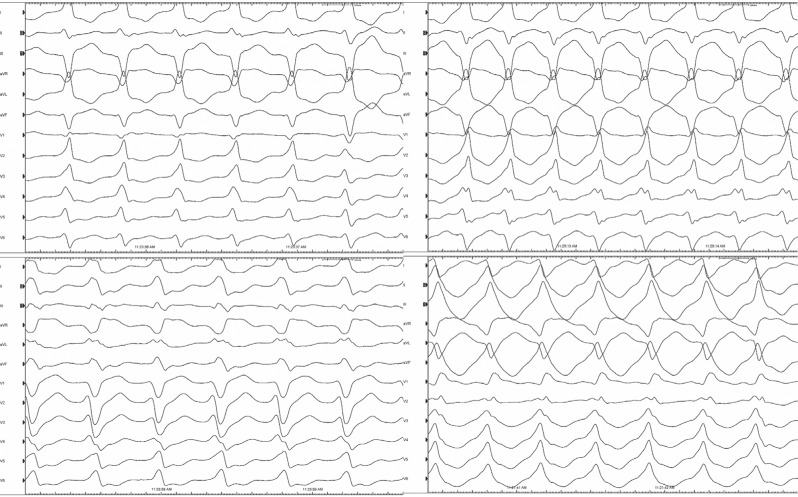
Four different ventricular tachycardia morphologies are seen.

Some associative and dissociative maneuvers for dissociating a certain electrical signal from others of known origin are used to ascertain the source of an unknown potential.^1,4,5^ The atrial extra-stimulus testing might give us some important clues. The timing of the extra potential is expected to correlate with either the degree of pre-excitation or the amount of fusion (if a PVC or fascicular beat occurs). If the candidate signal is ventricular or otherwise associated with pre-excitation (eg, AP potential), then causing a delay to signal activation would cause less pre-excitation.^2^ In the current case, there was a delay from the atrial signal to both the HB and the candidate potential without a change in the degree of pre-excitation an atrial extra-stimulus beat **(Figure 2)** that showed more decrement in conduction down the AVN. A relatively fixed relationship was seen with V to this potential (single arrows), with a further delay with S2 (double arrow) in activating the tissue responsible for the extra (unexplained) potential. However, it would be difficult to explain the phenomenon via the effects of atrial pacing with a Mahaim fiber.^2^ For this to occur, a very similar decrement in conduction down the Mahaim fiber and the AVN is needed. The delay to the extra potential despite AH prolongation excluded all AP-related diagnostic possibilities in the current case, including NV pathways.^6–8^ Electroanatomic mapping revealed periaortic fibrosis that was consistent with this unusual potential **(Figure 3)**, and then 4 different ventricular tachycardia (VT) morphologies were induced **(Figure 4)**.

Periaortic fibrosis is increasingly recognized as a distinct clinical substrate for scar-related VT in the presence and absence of overt structural heart disease.^9–11^ These VTs from small regions of the periaortic scar can mimic idiopathic VT but are suggested by multiple VT morphologies **(Figure 4)**.^9,12^ Several methods have been studied to invoke dynamic substrate changes in critical regions to target ablation, including decrement-evoked potential mapping,^4,5^ which involves a drivetrain and an S2 pacing protocol identifying sites of decremental LAVAs. This small periaortic site was then targeted for radiofrequency ablation **(Figure 3)**. During ablation, the amplitude of this potential decreased, and all VT morphologies were no longer seen. Electrophysiologists must have a thorough understanding of how unexplained potentials can be analyzed using pacing maneuvers and by observing changes of association and dissociation that occur between these unknown signals and other known potentials (ventricular, atrial, AP, LAVA, etc.).^1^ These techniques are useful in identifying the culprit sites and targets for ablation for several arrhythmias.^1,2^

## Figures and Tables

**Figure 1:**
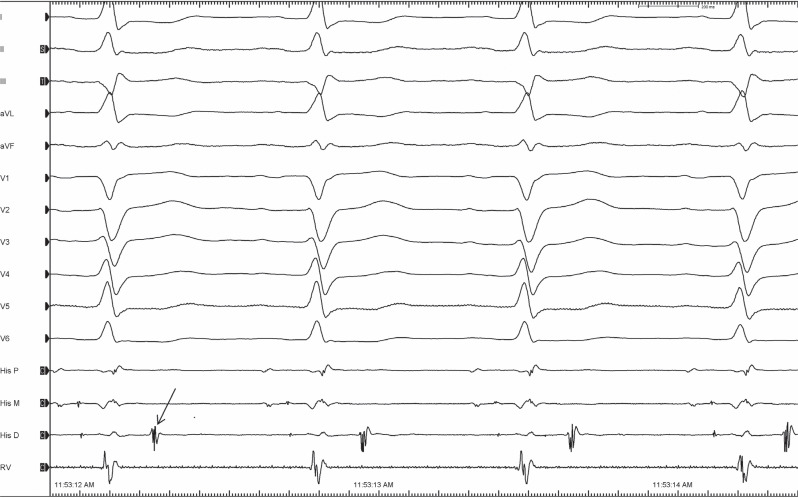
The extra potential is seen after the ventricular deflection on the His-bundle catheter (arrow) during sinus rhythm.
